# Consumer perception and sensory profile of probiotic yogurt with added sugar and reduced milk fat

**DOI:** 10.1016/j.heliyon.2020.e04328

**Published:** 2020-07-04

**Authors:** P.G.I. Dias, J.W.A. Sajiwani, R.M.U.S.K. Rathnayaka

**Affiliations:** Department of Food Science and Technology, Faculty of Applied Sciences, Sabaragamuwa University of Sri Lanka, P. O. Box 02, Belihuloya, Sri Lanka

**Keywords:** Food science, Food analysis, Added sugar, Milk fat, Yogurt

## Abstract

The objective of this study was to determine the effect of different milk fat (MF, 0%, 1.5%, 3.3% (w/w)) or added sugar (AS, 0%, 3.5%, 7% (w/w)) levels on the sensory properties and consumer acceptability of probiotic set yogurts fermented by *Streptococcus thermophilus*, *Bifidobacterium bifidum*, and *Lactobacillus delbrueckii subsp. bulgaricus*. The experimental yoghurt samples were compared with a control containing AS, 7% and MF, 3.3%. A quantitative descriptive analysis was conducted using 12 semi-trained panelists to evaluate the sensory profile of yogurts by rating the intensity of sensory descriptors on a 10 unit scale. Consumer preferences was determined using a group untrained individuals (n = 31) by applying the hedonic test for preference. Sensory mapping and Principal Component Analysis (PCA) were conducted to obtain a comprehensive understanding of the consumer behavior. Partial Least Squares Regression (PLSR) was conducted to compare the sensory profiling, consumer preference and instrumental data. Descriptive data showed significant differences (p < 0.05) among the samples for 8 of the 15 attributes amalyzed, including flavor and texture parameters. PCA showed that more than 80% of consumers highly preferred the low-fat yogurt, basically due to its firmness. Overall, consumer preference decreases with the AS reduction. The sample containing zero sugar is the least preferred sample. The reduction of added sugar below 3.5% is a challenge in terms of retaining product quality.

## 1 Introduction

Food trends of the worlds’ population have been largely altered during the last two decades due to the alarming rates of non-communicable diseases. People believe in the dietary management of such diseases over the costly medicinal drugs with possible side effects. Therefore, the demand for health-promoting or functional foods has increased significantly in recent times. Low/no fat, low/no added sugar and low/no-calorie are some popular claims under this functional food category (Sloan, 2018).

However, eliminating fat and/or added sugar from a food product is a challenge. They are not only responsible for adding calories but also can act as a bulking agent, natural preservative, tenderizer, colorant, anticoagulant, and texture improver in foods (Alexander, 1998). Similarly, fat is associated with melting, crystallization, texture formation, aeration, heat transfer, lubrication, pleasant appearance, and flavor of the food (Devi and Khatkar, 2016).

Therefore, eliminating these two components might alter the unique texture, taste, odor, appearance, color, shelf life, etc. of the food. How consumers perceive this alteration is highly doubtful. If they reject this change in their well -known food that will ultimately cause a huge loss in profit and disrepute to the manufacturing company. Therefore, knowledge of how fat and sugar elimination affect the sensory properties of a food product is a must.

Yogurt is the most popular dairy product worldwide (El Samh et al., 2013). Low/no added sugar and low/no milk fat containing yogurts have also been recently introduced to the market. However, studies on the impact of quality attributes of yogurt as milk fat and added sugar reduction were under-exploited (Kahkonen et al., 1997). The interaction effect of these two parameters towards yogurt was discussed by only a few scientists (Johansen et al., 2010). Therefore, the present study focused on assessing the consumer perception and sensory profile of yogurt with reduced amounts of added sugar and milk fat.

## 2 Methods

### 2.1 Sample preparation

For the experimental analysis, five yogurt samples were prepared in the Dairy Processing Laboratory, Department of Food Science and Technology, Sabaragamuwa University of Sri Lanka (Table 1). Yogurt samples were prepared from no fat, low fat, and full cream UHT treated milk (Kotmale Dairy Products (Pvt) Ltd, Sri Lanka), and the fat contents were tested and confirmed by the Gerber method (Kleyn et al., 2001).

**Table 1:** Combination of milk fat and added sugar in each yogurt sample.

Milk fat % (w/w)	Added sugar % (w/w)	Sample Tag
3.3	7	C (control)
3.3	3.5	LS (low sugar)
3.3	0	NS (no sugar)
1.5	7	LF (low fat)
0	7	NF (no fat)

Samples were prepared with different combinations of fat and sugar as given in Table 1.

### 2.2 Consumer preference test

A consumer preference test was conducted with 31 consumers between the ages of 20–50 years. Written consent was taken from those consumers for their participation before starting the test, and ethical clearance was obtained from the Ethical Review Committee of the Sabaragamuwa University of Sri Lanka. Each consumer rated the five-set yoghurt samples on a 5-point hedonic scale (1 strongly dislike to 5 strongly like) for seven liking attributes (appearance, color, odor, texture, taste, aftertaste, overall acceptability). The five-set yogurt samples were served to the consumer in 20 mL plastic cups covered with lids. Water and unsalted crackers were provided for palate cleansing. Each sample was labeled with a random 3-digit code and served in a random order to each consumer. A fresh batch of each of the five-set yogurt samples was prepared for the consumer test.

### 2.3 Descriptive sensory analysis

Five yogurt samples were evaluated by a trained sensory panel using a line scale with labelled endpoints going from no intensity (value 1.0) at the left side to high intensity (value 10.0) at the right side. The panel composed of fourth-year students from the Department of Food Science and Technology, Sabaragamuwa University of Sri Lanka. Twelve panelists were selected and trained according to ISO standards (ISO, 1993), in the sensory laboratory set up according to ISO standards (ISO, 1988). Written consent was taken from the panelists for their participation before the start of the test, and ethical clearance was obtained from the Ethical Review Committee of the Sabaragamuwa University of Sri Lanka. After panel training, the yogurt samples were served in random order to each panelist. Yogurt samples were freshly prepared and kept in a refrigerator until serving. The panelists rinsed their mouths with water before testing each sample and unsalted cracers were used for palate cleansing.

### 2.4 Statistical analysis

Statistical analysis was conducted by the one-way ANOVA method using Minitab 17 statistical software (Minitab Inc, USA) for the consumer preference data. Sensory mapping, Principal Component Analysis (PCA) and Partial Least Square Regression (PLSR) analysis of descriptive sensory data were conducted by XLSTAT (V5.2; Addinsoft, Paris, France) software. PCA and PLS were selected based on the features listed below.

Principal Component Analysis (PCA) is a multivariate statistical analytical technique applied to data to reduce the set of dependent variables (attributes) to a smaller set of underlying variables (called factors) based on patterns of correlation among the original variables (Lawless and Heymann, 2010). Partial Least Squares (PLS) is an extension of PCA which is used for relating the variations in one or several response variables (Y variables or dependent variables) to the variations of several predictors (X variables) (Long, 2013).

## 3 Results and discussion

### 3.1 Consumer preference

According to the present study, no significant difference (p < 0.05) exist for the consumer ranking of appearance, color, odor, and texture (Table 2). However, instrumental texture profile analysis showed a significant difference in five samples for texture parameters. Instrumental data for color values also tally with these results.

**Table 2:** Average rank values of consumer preference data.

Sample	Average rank value for each attribute						
	Appearance	Colour	Odor	Texture	Taste	After Taste	Overall acceptability
C	4±1^a^	4±1^a^	4±1^a^	3±1^a^	4±1^a^	4±1^a^	3±1^b^
LS	4±1^a^	4±1^a^	3±1^a^	4±1^a^	3±1^b^	3±1^b^	3±1^c^
NS	4±1^a^	4±1^a^	3±1^a^	4±1^a^	2±1^c^	2±1^c^	2±1^d^
LF	4±1^a^	4±1^a^	4±1^a^	4±1^a^	4±1^a^	4±1^a^	4±1^a^
NF	4±1^a^	4±1^a^	4±1^a^	3±1^a^	4±1^a^	4±1^a^	4±1^ab^

Mean ± SE (n = 3). Values followed by different letters in the same column are significantly different (p < 0.05) according to Tukey's test.

As expected, low sugar and no sugar yogurts expressed the least preference for taste and after taste (Table 2). The results are in accordance with the findings of Johansen et al. (2010). Another study showed a bitter after taste in ice creams after reducing fat and sugar (Cadena et al., 2012). In this study, the sour taste of yogurt is dominating with increasing sugar reduction, in agreement with previous findings (Harper et al., 1991).

There was an interaction effect on sweetness (added sugar content) and richness (milk fat content) on consumer perception (Johansen et al., 2010). Previous studies showed that fat in the food matrix was highly infleuential for sweet taste perception (McCain et al., 2018).

**Figure 1:**
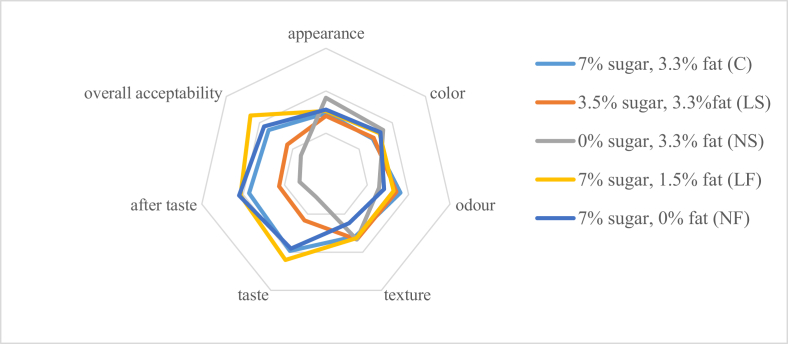
Radar chart of consumer preference data.

According to Table 2 and Figure 1, the highest overall acceptability was observed in low-fat yogurt, followed by no fat and full cream yogurts. Low sugar and no sugar yogurts have significantly (p < 0.05) less overall acceptability (Table 2). The higher sugar content of yogurts leads to higher overall liking which is in agreement with the findings of Hoppert et al. (2013). Accordingly, reduction of added sugar up to 3.5% and below was not accepted by the consumers, yet fat reduction is accepted. Several studies also revealed that yogurt containing 7% sugar is accepted by the consumer panel (Chollet et al., 2013). Similar findings came out from the present study, while 3.5% and 0% added sugar contents were unacceptable. According to a survey conducted in the UK, 55% of low-fat yogurts contained between 10 and 20 g sugar/100 g (Moore et al., 2018). According to this study, there is a potential to reduce total sugar up to 7% (w/w).

### 3.2 Descriptive sensory analysis

**Figure 2:**
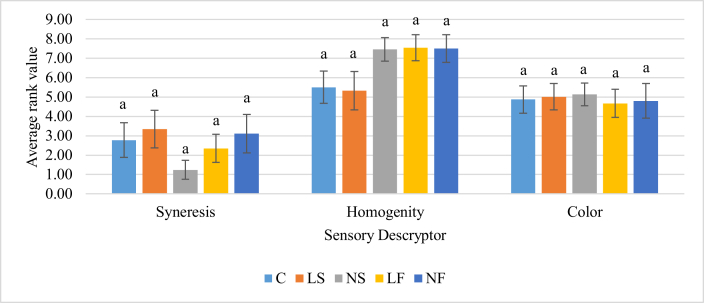
Average rank values for appearance-related sensory descriptors. Values followed by different letters in the bars in the same attribute are significantly different (p < 0.05) according to Tukey's test.

Results of descriptive sensory analysis showed that there are no significant differences among the samples for color, syneresis, and homogeneity (Figure 2). This is in contrast with the instrumental data for syneresis. However, the color values tallied with consumer and instrumental data.

**Figure 3:**
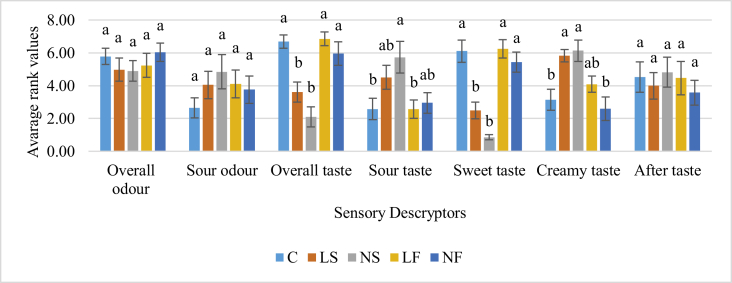
Average rank values for taste and odor related sensory descriptors. Values followed by different letters in the bars in the same attribute are significantly different (p < 0.05) according to Tukey's test.

According to the studies of Brauss et al. (1999), low-fat yogurts (0.2%) were found to release volatiles more quickly and at a higher intensity, but with less persistence than yogurts containing fat at 3.5 and 10%. In contrast, no significant difference in odor parameters was observed in the present study (Figure 3).

Accordingly, taste perception significantly (p < 0.05) reduces with the sugar reduction but does not influence the after taste (Figure 3).

**Figure 4:**
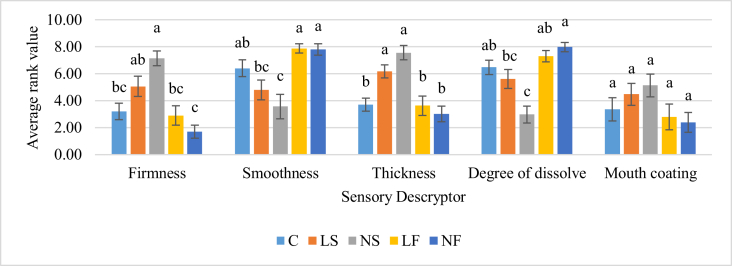
Average rank values for texture related sensory descriptors. Values followed by different letters in the bars in the same attribute are significantly different (p < 0.05) according to Tukey's test.

As described by Frost and Janhoj (2007), texture perception, particularly smoothness and creaminess of low-fat dairy products, seems to be the driving factor for consumer acceptance. In this study, texture parameters were largely influenced by both milk fat and added sugar content. No sugar (0% sugar and 3.3% fat combination) showed the highest thickness, firmness and mouth coating and lowest smoothness and degree of dissolution followed by the low sugar combination (3.5% sugar and 3.3% sugar) and then the high sugar, full cream control (7% sugar, 3.3% fat) sample (Figure 4). Similar trends were observed in texture profile analysis and rheological data as well (Instrumental data). However, the highest total solid content was observed in the control sample. Therefore, more evidence needs to explain this unexpected variation.

The study of Baines and Morris (1987) had revealed ‘at higher viscosities, it is harder to perceive sweet taste, so thicker foods require higher concentrations of sugar to obtain equivalent sweetness. High-fat contents also act in a similar manner (Malone et al., 2003). In that sense, sugar reduction is far more challenging without reducing the fat content.

### 3.3 Sensory mapping and Principal Component Analysis (PCA)

**Figure 5:**
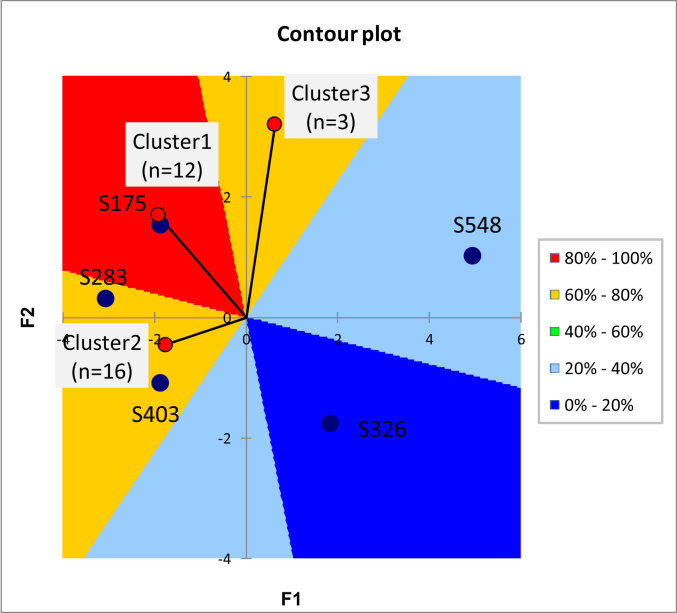
Contour plot for consumer preference and sensory profile of five samples. Sample codes 403, 326, 548, 175, and 283 stand for C; LS; NS; LF; and NF, respectively.

**Figure 6:**
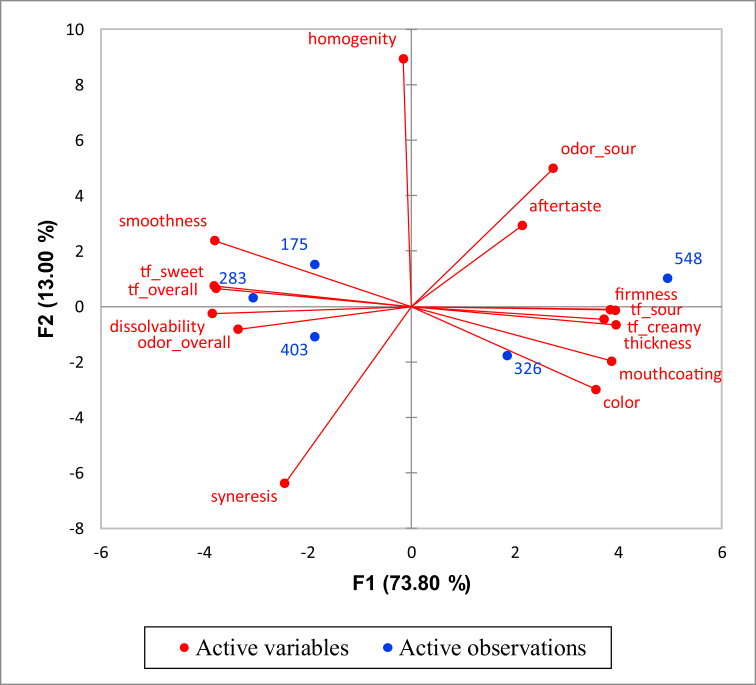
Principal Component Analysis (PCA) for consumer preference and sensory profile of five samples. Sample codes 403, 326, 548, 175, and 283 stands for C; LS; NS; LF; and NF, respectively.

Contour plot for consumer preference (Figure 5) and Principal Component Analysis (PCA) (Figure 6) revealed the direction and intensity of descriptive sensory attributes for yogurts with different milk fat and added sugar contents and allowed for comparison of descriptive sensory profiling data of each yogurt combination.

The contour plot of consumer preference for the yogurt samples is shown in Figure 5. Three clusters of consumers were identified based on preference for the samples. Vector models were obtained for all three clusters with no saddle or ideal points. Consumers in cluster 1 displayed a high preference for sample coded as S175 (7% sugar and 1.5% fat; LF) while their preference for sample coded as S326 (3.5% sugar, 3.3% fat; C) was low. Consumers in cluster 2 preferred samples coded as S283 (7% sugar and 0% fat; NF) and S403 (7% sugar and 3.3% fat; C) but had a low preference for sample coded as S548 (0% sugar and 3.3% fat; NS). But cluster 3 did not have a specific preference, probably due to the low number of consumers in this cluster (Figure 5).

In PCA, the first dimension (F1, 73.80% variance explained) variance explained was mainly associated with thickness, mouth-coating, color, creamy taste (tf_cremy), and sour taste (tf_sour). These attributes were opposed by smoothness, sweet taste (tf_sweet), overall taste (tf_overall), dissolvability, and overall odor (odor_overall). The second dimension (F2, 13% variance explained) was mainly associated with homogeneity. That attribute is opposed by syneresis (Figure 6).

Preference for sample coded as S175 (7% sugar and 1.5% fat; LF) was related to the smoothness attribute. Samples coded as S283 (7% sugar and 0% fat; NF) and S403 (7% sugar and 3.3% fat; C) in cluster 2 were characterized by the sweet taste, overall taste, dissolvability, and overall odor (Figure 5 and Figure 6).

Samples coded as S548 (0% sugar and 3.3% fat; NS) and S326 (3.5% sugar, 3.3% fat; LS) showed the lowest numbers of consumers whose preferences calculated from the model were higher than the mean preference (in other words, these samples had low preference scores from the consumers). These samples were characterized by levels of firmness, sour taste, creamy taste, thickness, mouth coating, and color that consumers did not like (Figure 5 and Figure 6).

### 3.4 Comparison of instrumental, sensory profiling and consumer preference data

**Figure 7:**
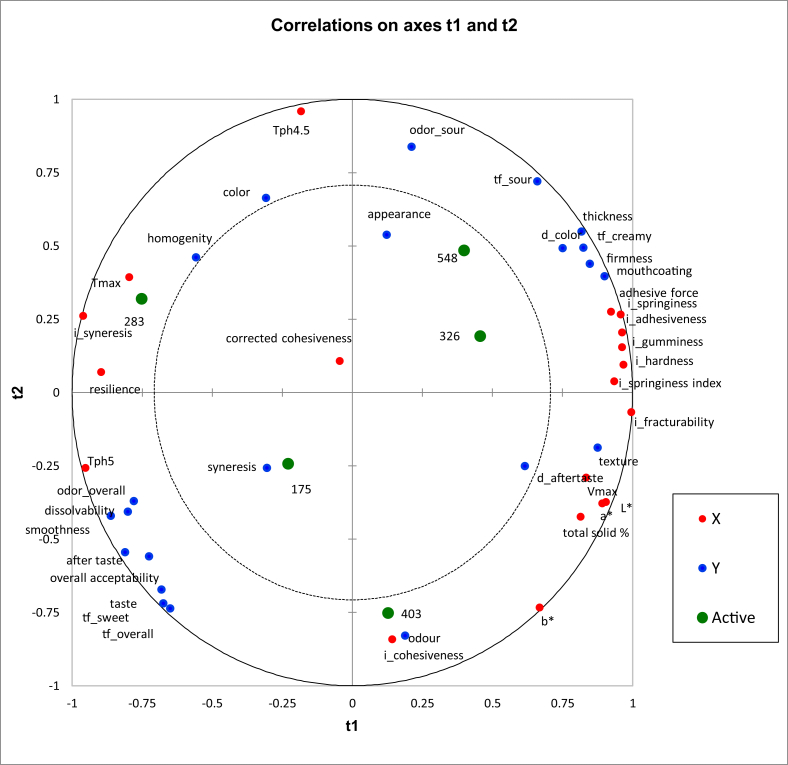
Partial Least Square Regression (PLSR) biplot correlating consumer preference, sensory profile (blue dots), and instrumental data (red dots) of five samples (green dots). Sample codes 403, 326, 548, 175, and 283 stands for C; LS; NS; LF; and NF, respectively.

Partial Least Squares Regression (PLSR) is often used when there are a lot of explanatory variables, possibly correlated. Here, PLSR analysis was used to predictively model the relationship between sensory profiling, consumer acceptances, and instrumental data such as fermentation kinetic values, color values, and texture profile analysis. PLSR showed (Figure 7) that, adhesive force, springiness, gumminess, adhesiveness, hardness, springiness index obtained from texture analyzer (Brookfield CT3) are positively correlated with descriptive sensory analysis attributes of thickness, firmness, mouth-coating, white color (d_color) and creamy flavor (tf_creamy). Creaminess, thickness, adhesiveness, and mouth-coating higher in full cream yogurts. The white color of full cream yogurt being higher than the no-fat yogurt may be due to the light scattering by fat globules. In that sense, this positive correlation is acceptable. Sour taste (tf_sour) and sour odor (odour_sour) data from the descriptive sensory analysis are negatively correlated with the overall acceptability of consumer preference data. Further, this overall acceptability is positively correlated with taste and after taste in consumer preference data and smoothness, dissolvability, overall odor (odor_overall), overall taste (tf_overall), and sweet taste (tf_sweet) from descriptive sensory data. Sour odor (odor_sour) in sensory profile data is negatively correlated with the odor parameter of consumer preference data. Total solid % has a positive correlation with texture attribute in consumer preference data. However, some relationships between the attributes is hard to explain e.g., the positive correlation between total solid %, V_max_ and a∗, b∗ values.

The reduction of milk fat and added sugar contents have an individual effect on consumer perception. Descriptive data showed significant differences (p < 0.05) among the samples for 8 of the 15 attributes, including flavor and texture parameters. Overall, consumer preference reduces with the AS reduction. Zero sugar is the least preferred sample. Thus, the reduction of added sugar up to or below 3.5% is a challenge. PCA showed more than 80% of consumers highly preferred to low-fat yogurt, basically due to its firmness. Positive and negative relationships were observed between instrumental, sensory profiling, and consumer preference data of sensory attributes. However, some relationships between attributes are hard to explain. Further research needs to be conducted to explain the high firmness of low-fat yogurt over the full cream sample.

## 4 Conclusion

The effect of the reduction of added sugar and milk fat on the consumer acceptance of probiotic yogurt was studied and significant differences among the samples were observed for 8 of the 15 attributes tested, including flavor and texture parameters. PCA showed that more than 80% of consumers highly preferred the low-fat yogurt, basically due to its firmness. Overall, consumer preference reduces with the added sugar reduction. Unhealthy fats and added sugars are prominent risk factors for diet-related non-communicable diseases. Hence, reshaping the food recipes by reducing, eliminating or replacing those is a timely requirement. According to the results it can be concluded that it is possible to reduce the fat content of yogurt as it increased the consumer acceptance. However, as reduction of sugar negatively affected the consumer acceptance, further studies are needed to investigate other types of sweeteners suitable to replace the sugars.

## Declarations

### Author contribution statement

Imanthika Dias: Conceived and designed the experiments; Performed the experiments; Analyzed and interpreted the data; Wrote the paper.

Amanda Sajiwanie: Conceived and designed the experiments; Performed the experiments; Analyzed and interpreted the data.

Udaya Rathnayaka: Conceived and designed the experiments; Performed the experiments; Analyzed and interpreted the data; Contributed reagents, materials, analysis tools or data.

### Funding statement

This work was supported by 10.13039/501100011609Sabaragamuwa University of Sri Lanka, Sri Lanka [SUSL/RG/2017/05].

### Competing interest statement

The authors declare no conflict of interest.

### Additional information

No additional information is available for this paper.
